# Impact of statin use on short- and long-term outcomes in patients with sepsis-induced myocardial injury: insights from the MIMIC-IV database

**DOI:** 10.3389/fphar.2025.1520107

**Published:** 2025-03-19

**Authors:** Yuan Liu, Jijiang Chen, Yehao Yuan, Pingping Niu, Mengyi Wu, Baoling Shang, Weihui Lu, Xu Zou, Gengzhen Yao

**Affiliations:** ^1^ Second Clinical Medical College, Guangzhou University of Chinese Medicine, Guangzhou, China; ^2^ The Second Affiliated Hospital of Guangzhou University of Chinese Medicine, Guangdong Provincial Hospital of Chinese Medicine, Guangzhou, China

**Keywords:** statin, sepsis-induced myocardial injury, all-cause mortality, intensive care unit, MIMIC-IV database

## Abstract

**Background:**

Sepsis-induced myocardial injury (SIMI) is a critical complication of sepsis, marked by high mortality rates, and lacks effective treatments. The impact of statin therapy on mortality in SIMI patients remains unclear. This study aims to explore the association between statin use and mortality in SIMI patients, focusing on both short-term and long-term outcomes.

**Methods:**

A retrospective cohort study was conducted by extracting SIMI patient information from the Medical Information Mart for Intensive Care IV (MIMIC-IV) database. Patients were categorized into statin and non-statin groups. A 1:1 nearest propensity-score matching (PSM) was used to balance baseline characteristics. Survival outcomes were assessed using Kaplan-Meier analysis and robust Cox proportional hazards models to understand the effects of statin use, type and dosage on mortality at 28 days, 90 days, and 1 year. E-Value analysis was used for unmeasured confounding.

**Results:**

A total of 2,246 patients meeting SIMI criteria were enrolled in the final cohort, with 17.9% receiving statins during their ICU stay. Statin use was associated with significantly lower mortality at all time points, as shown by Kaplan-Meier analysis. In multivariable robust Cox regression models, statin therapy correlated with a 32% reduction in 28-day mortality (HR = 0.68, 95% CI: 0.49–0.94), a 29% reduction at 90 days (HR = 0.71, 95% CI: 0.54–0.93), and a 28% reduction at 1 year (HR = 0.72, 95% CI: 0.58–0.90), maintaining significance after adjustment for confounders. Simvastatin was particularly effective, and low-dose statins were linked to reduced mortality risk. Subgroup analyses suggested consistent statin benefits. E-Value analysis suggested robustness to unmeasured confounding.

**Conclusion:**

Our study demonstrates that statin use is significantly associated with reduced mortality in SIMI patients across 28 days, 90 days, and 1 year. Simvastatin provides substantial benefits, with low-dose statins providing greater advantages compared to high-dose formulations.

## 1 Introduction

Sepsis affects millions globally, accounting for approximately 30.2% of intensive care unit (ICU) admissions, and is a leading cause of mortality due to the organ dysfunction it induces (Singer et al., 2016; Machado et al., 2017). Among these complications, sepsis-induced myocardial injury (SIMI) is particularly critical, characterized by high morbidity and mortality rates. Previous studies have reported a wide prevalence of septic cardiomyopathy, ranging from 10% to 70% (Beesley et al., 2018). Although SIMI is often considered a reversible complication of sepsis, the prognosis for affected patients remains grim, with an in-hospital mortality rate of 35% and a 1-year mortality rate of 51% (Frencken et al., 2018). The pathophysiology of SIMI involves complex interactions between inflammatory responses and myocardial dysfunction (Hollenberg and Singer, 2021), which contribute to these elevated mortality rates. Despite advancements in sepsis management, effective treatments for SIMI remain elusive, underscoring the urgent need for novel therapeutic strategies.

Statins, 3-hydroxy-3-methylglutaryl-coenzyme A (HMG-CoA) reductase inhibitors, are well-known for their lipid-lowering effects, which include decreased serum total cholesterol (TC), low-density lipoprotein (LDL), reduced triglycerides (TG), and a modest increase in high-density lipoprotein (HDL) (Barter et al., 2010; Ferraro et al., 2022). In addition, statins exhibit pleiotropic effects, including anti-inflammatory properties and improvements in endothelial function, suggesting potential benefits in inflammatory and vascular conditions (Koushki et al., 2021; Mollazadeh et al., 2021; Li B. et al., 2024). Recent studies have explored the role of statins in sepsis and heart failure due to their cardioprotective effects (Li M. et al., 2024; Zheng et al., 2024). However, the implications of statin therapy for SIMI remain poorly understood.

Despite these promising insights, the prognostic value of statin therapy in SIMI remains uncertain. There is a critical need to delineate the role of statins in improving outcomes for SIMI patients, which could potentially transform therapeutic approaches in SIMI management. Our study utilized the MIMIC-IV database to investigate the association between statin use and outcomes in SIMI patients.

## 2 Materials and methods

### 2.1 Data sources

In this retrospective cohort study, we extracted the data from the Medical Information Mart for Intensive Care (MIMIC)-IV database, found at https://physionet.org/content/mimiciv/3.0/. This extensive, single-center medical database, freely available to the public, is maintained by Beth Israel Deaconess Medical Center and boasts a rich patient data collection. It documented over 360,000 emergency department admissions and more than 90,000 ICU stays from 2008 to 2022 (Johnson et al., 2023). In alignment with the Collaborative Institutional Training Initiative, one of our authors completed an examination (certification number 65786107 for author Yuan Liu).

### 2.2 Study population

The criteria for participant inclusion were defined by several key factors: (1) participants had to be 18 years of age or older, (2) they needed to fulfill the Sepsis 3.0 criteria (Singer et al., 2016), (3) they had to meet the SIMI criteria, (4) their ICU stay had to be more than 24 h but not exceed 100 days. We also considered direct or indirect causes that could lead to an abnormal release of cardiac troponin T (cTnT), which included conditions such as acute coronary syndrome, cardiomyopathy, myocarditis, valvular disease, endocarditis, pericarditis, chronic obstructive pulmonary disease, chronic heart failure, a history of prior cardiac surgery or cardiac arrest before being admitted to the ICU, and a history of severe tachyarrhythmia, including supraventricular tachycardia, ventricular tachycardia, ventricular fibrillation, and ventricular flutter. In addition, we also excluded patients with reduced clearance due to combined chronic kidney disease.

### 2.3 Data collection

Structured Query Language was used to extract data in Navicat Premium software (version 16) based on the stay_id and hadm_id of patients (Wu et al., 2021). Clinical information was extracted from the MIMIC-IV database for the first record after admission to the ICU for SIMI patients, including demographic characteristics, biochemical indicators, clinical scores, treatments, and medication information. We also extracted comorbidities, length of ICU stay, length of hospital stay, and mortality outcomes, with comorbidity derived from the ICD code recorded for the patient’s discharge diagnosis. Detailed information on statin use included the drug name, type, dosage, and time of administration.

### 2.4 Diagnosis of SIMI

All cTnT results were extracted from tests performed after the patient was admitted to the ICU, and the SIMI assessment was performed using the initial value. The 99th percentile of the reference upper limit for cTnT in this center is 0.01 ng/mL, and SIMI is defined as cTnT >0.01 ng/mL (Vallabhajosyula et al., 2017; Dong et al., 2024).

### 2.5 Outcomes of this study

The primary endpoint of this study was at 1-year mortality. Secondary outcomes included 28-day mortality and 90-day mortality.

### 2.6 Propensity score matching (PSM)

PSM was utilized to adjust for covariates, ensuring the robustness of our study results (Zhang, 2017). We employed an algorithm of 1:1 nearest neighbor matching with a caliper width of 0.05 for this analysis. The variables included in the propensity score model were chosen based on the statistical differences observed between the two groups at baseline and consensus statements published in the literature (Cecconi et al., 2018). These variables encompassed age, heart rate, systolic blood pressure (SBP), peripheral capillary oxygen saturation (SpO2), the presence of diabetes, atrial fibrillation, cirrhosis, hyperlipidemia, hypertension, levels of cTnT, platelet (PLT), lactic acid (LAC), as well as sequential organ failure assessment score (SOFA), simplified acute physiology score II (SAPS II), logistic organ dysfunction system score (LODS), and the receipt of treatments including Continuous Renal Replacement Therapy (CRRT), Vasopressin, Angiotensin-Converting Enzyme Inhibitor (ACEI), Angiotensin II Receptor Blocker (ARB), Aspirin, Beta-blockers, and Fibrates.

### 2.7 Statistical analysis

Patients were categorized into non-statin group and statin group based on their use of statins. To minimize bias caused by missing data, we deleted variables with more than 30% missing data, and used the random forest method in the mice package (version 3.16.0) to perform multiple imputation for variables with a missing value rate of less than 30% (Buuren and Groothuis-Oudshoorn, 2011). The percentages of variables are shown in Supplementary Table S1. Continuous variables with normal distribution were expressed as mean ± standard deviation, while those with non-normal distribution were represented by interquartile ranges. Student's t-tests and Wilcoxon rank-sum tests were used to assess the significance of differences between the statin and non-statin groups. Categorical variables were presented as frequencies and percentages, with differences between the two groups assessed using chi-square tests.

We utilized Kaplan-Meier analysis to calculate the 28-day, 90-day, and 1-year survival probabilities for patients in the statin and non-statin groups. The impact of statin therapy on mortality was evaluated using robust Cox proportional hazards models, with hazard ratios (HR) and 95% confidence intervals (CI). Three robust Cox proportional hazards regression models were constructed with patients in the non-statin group as the reference: (1) Model I, an unadjusted model; (2) Model II, which included age, male, race, heart rate, SBP, DBP, RR, SpO2, weight, diabetes, atrial fibrillation, cirrhosis, hyperlipidemia, hypertension, obesity; (3) Model III, which added cTnT, creatine kinase-MB (CK-MB), hemoglobin (Hb), PLT, White Blood Cell Count (WBC), Blood Urea Nitrogen (BUN), Serum Creatinine (SCR), LAC, SOFA, SAPS II, LODS, Invasive Mechanical Ventilation (IMV), CRRT, vasopressin, ACEI, ARB, aspirin, beta-blockers, fibrates. We also evaluated the correlation between the type and dosage of statins and mortality in the three models.

Subgroup analyses were conducted to investigate whether demographic characteristics and comorbidities affected the correlation between statin administration and 28-day, 90-day and 1-year mortality. We also explored potential unmeasured confounding factors between statin use and mortality in patients with SIMI by calculating the E-Value (Haneuse et al., 2019). The E-Value quantifies the magnitude of an unmeasured confounder that would be necessary to negate the observed effect of statins on mortality in SIMI patients. All statistical analyses for our study were conducted utilizing R software (v.4.3.1). A significance level of P < 0.05 was considered statistically significant.

## 3 Results

### 3.1 Baseline characteristics of patients SIMI

**FIGURE 1:**
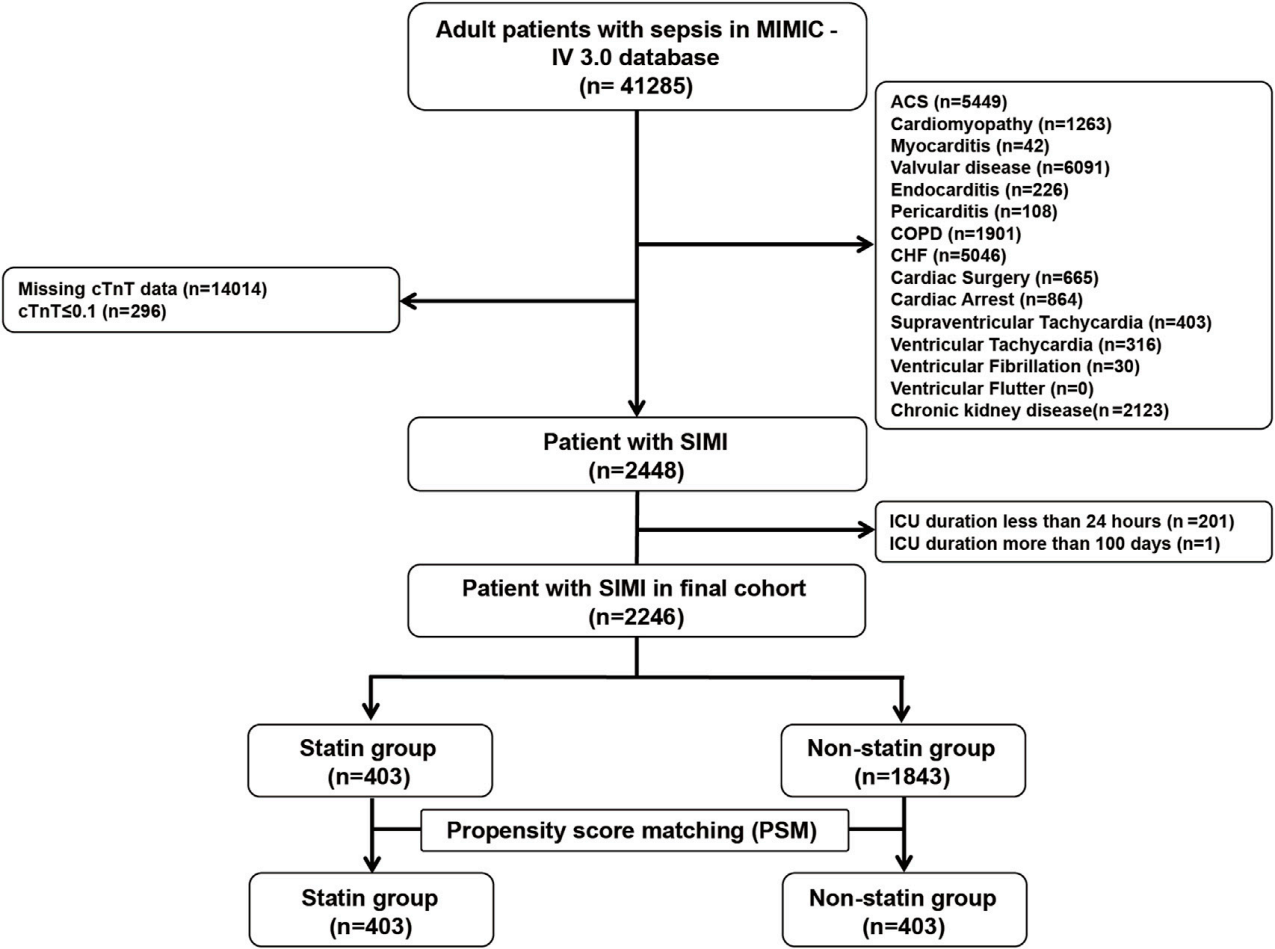
Flow chart of the cohort selection process.

A total of 2,246 patients meeting SIMI criteria were enrolled in the cohort, and the detailed flow was shown in Figure 1. In this cohort, 17.9% of patients (n = 403) were given statins during their ICU stay. Compared with the non-statin group, patients in the statin group were older, had lower heart rates, high systolic blood pressure and SPO2, lower lactate levels but higher cTnT and PLT levels, lower prevalence of cirrhosis, higher prevalence of diabetes mellitus and other chronic comorbidities (Atrial Fibrillation, hyperlipidemia), and lower SOFA, SAPS II, and LODS scores. After PSM, a total of 806 patients (403 in each group) were for further study, and most of the baseline characterization biases were corrected (Table 1). A comparison of the standard mean difference before and after PSM is shown in Supplementary Figure S1.

**TABLE 1:** Baseline characteristics between groups before and after PSM.

Characteristic	Original cohort			PSM cohort		
	Non-statin group	Statin group	*P* ^a^	Non-statin group	Statin group	*P* ^1^
	N = 1,843	N = 403		N = 403	N = 403	
Demographic variables						
Age, Median (Q1, Q3)	65.00 (53.00, 76.00)	70.00 (63.00, 79.00)	<0.001	71.00 (62.00, 82.00)	70.00 (63.00, 79.00)	0.195
Male, n (%)	1,071.00 (58.11%)	209.00 (51.86%)	0.022	209.00 (51.86%)	209.00 (51.86%)	>0.999
Ethnicity, n (%)			0.175			0.437
WHITE	1,114.00 (60.44%)	241.00 (59.80%)		255.00 (63.28%)	241.00 (59.80%)	
BLACK	233.00 (12.64%)	64.00 (15.88%)		52.00 (12.90%)	64.00 (15.88%)	
Other	496.00 (26.91%)	98.00 (24.32%)		96.00 (23.82%)	98.00 (24.32%)	
Vital signs on admission, Median (Q1, Q3)						
HR (beats/min)	76.00 (65.00, 87.00)	70.00 (60.00, 81.00)	<0.001	71.00 (61.00, 81.00)	70.00 (60.00, 81.00)	0.494
SBP (mmHg)	85.00 (76.00, 94.00)	88.00 (78.00, 97.00)	0.006	86.00 (77.00, 99.00)	88.00 (78.00, 97.00)	0.636
DBP (mmHg)	44.00 (37.00, 51.00)	44.00 (37.00, 51.00)	0.521	43.00 (37.00, 50.00)	44.00 (37.00, 51.00)	0.844
RR (beats/min)	13.00 (10.00, 16.00)	12.50 (10.00, 15.00)	0.132	13.00 (10.00, 15.00)	12.50 (10.00, 15.00)	0.499
SpO2 (%)	92.00 (90.00, 95.00)	93.00 (90.00, 95.00)	0.044	92.00 (90.00, 95.00)	93.00 (90.00, 95.00)	0.286
Weight (kg)	78.00 (65.20, 93.70)	77.30 (66.20, 91.70)	0.931	76.30 (63.80, 91.00)	77.30 (66.20, 91.70)	0.22
Comorbidity, n (%)						
Diabetes	494.00 (26.80%)	178.00 (44.17%)	<0.001	175.00 (43.42%)	178.00 (44.17%)	0.831
Atrial Fibrillation	424.00 (23.01%)	121.00 (30.02%)	0.003	132.00 (32.75%)	121.00 (30.02%)	0.404
Cirrhosis	329.00 (17.85%)	22.00 (5.46%)	<0.001	20.00 (4.96%)	22.00 (5.46%)	0.751
Hyperlipidemia	463.00 (25.12%)	217.00 (53.85%)	<0.001	204.00 (50.62%)	217.00 (53.85%)	0.359
Hypertension	677.00 (36.73%)	169.00 (41.94%)	0.051	221.00 (54.84%)	169.00 (41.94%)	<0.001
Obesity	153.00 (8.30%)	40.00 (9.93%)	0.292	33.00 (8.19%)	40.00 (9.93%)	0.39
Biochemistry, Median (Q1, Q3)						
cTnT (ng/ml)	0.06 (0.03, 0.12)	0.06 (0.03, 0.16)	0.008	0.05 (0.03, 0.13)	0.06 (0.03, 0.16)	0.096
CK-MB (IU/L)	4.00 (3.00, 9.00)	5.00 (3.00, 9.00)	0.643	4.00 (3.00, 8.00)	5.00 (3.00, 9.00)	0.422
Hb (g/dL)	9.30 (8.20, 10.70)	9.50 (8.30, 10.60)	0.322	9.60 (8.50, 10.80)	9.50 (8.30, 10.60)	0.102
PLT (K/uL)	179.00 (103.00, 288.00)	204.00 (143.00, 296.00)	<0.001	199.00 (143.00, 302.00)	204.00 (143.00, 296.00)	0.846
WBC (K/uL)	10.10 (7.10, 14.90)	9.50 (7.30, 13.50)	0.102	10.10 (7.50, 13.80)	9.50 (7.30, 13.50)	0.265
BUN (mg/dL)	23.00 (14.00, 39.00)	22.00 (15.00, 35.00)	0.373	22.00 (14.00, 35.00)	22.00 (15.00, 35.00)	0.538
SCR (mg/dL)	1.10 (0.70, 2.00)	1.10 (0.80, 1.80)	0.594	1.00 (0.70, 1.60)	1.10 (0.80, 1.80)	0.003
LAC (mmol/L)	2.00 (1.30, 3.20)	1.70 (1.20, 2.50)	<0.001	1.70 (1.20, 2.60)	1.70 (1.20, 2.50)	0.927
Critical assessment on admission, Median (Q1, Q3)						
SOFA	7.00 (4.00, 10.00)	6.00 (4.00, 8.00)	<0.001	6.00 (3.00, 8.00)	6.00 (4.00, 8.00)	0.86
SAPS II	43.00 (34.00, 54.00)	41.00 (33.00, 50.00)	0.004	42.00 (34.00, 51.00)	41.00 (33.00, 50.00)	0.269
LODS	6.00 (4.00, 9.00)	5.00 (4.00, 7.00)	<0.001	5.00 (4.00, 8.00)	5.00 (4.00, 7.00)	0.382
Treatment, n (%)						
IMV	1,177.00 (63.86%)	241.00 (59.80%)	0.126	244.00 (60.55%)	241.00 (59.80%)	0.829
CRRT	257.00 (13.94%)	28.00 (6.95%)	<0.001	31.00 (7.69%)	28.00 (6.95%)	0.685
Vasopressin	356.00 (19.32%)	39.00 (9.68%)	<0.001	38.00 (9.43%)	39.00 (9.68%)	0.905
Medication, n (%)						
ACEI	271.00 (14.70%)	103.00 (25.56%)	<0.001	106.00 (26.30%)	103.00 (25.56%)	0.809
ARB	19.00 (1.03%)	13.00 (3.23%)	<0.001	13.00 (3.23%)	13.00 (3.23%)	>0.999
Aspirin	302.00 (16.39%)	235.00 (58.31%)	<0.001	219.00 (54.34%)	235.00 (58.31%)	0.256
Beta blockers	752.00 (40.80%)	242.00 (60.05%)	<0.001	245.00 (60.79%)	242.00 (60.05%)	0.829
Fibrates	216.00 (11.72%)	72.00 (17.87%)	<0.001	69.00 (17.12%)	72.00 (17.87%)	0.781

^a^ Wilcoxon rank sum test; Pearson’s Chi-squared test.

Abbreviations: HR, Heart Rate; SBP, Systolic Blood Pressure; DBP, Diastolic Blood Pressure; RR, Respiratory Rate; SpO2, Peripheral Capillary Oxygen Saturation; WBC, White Blood Cell Count; cTnT, Cardiac Troponin T; CK-MB, Creatine KSinase-MB; Hb, Hemoglobin; PLT, Platelet Count; BUN, Blood Urea Nitrogen; SCR, Serum Creatinine; LAC, Lactic Acid; SOFA, Sequential Organ Failure Assessment; SAPSII, Simplified Acute Physiology Score II; LODS, Logistic Organ Dysfunction System; IMV, Invasive Mechanical Ventilation; CRRT, Continuous Renal Replacement Therapy; ACEI, Angiotensin-Converting Enzyme Inhibitor; ARB, Angiotensin II Receptor Blocker.

### 3.2 Kaplan-Meier analysis

**FIGURE 2:**
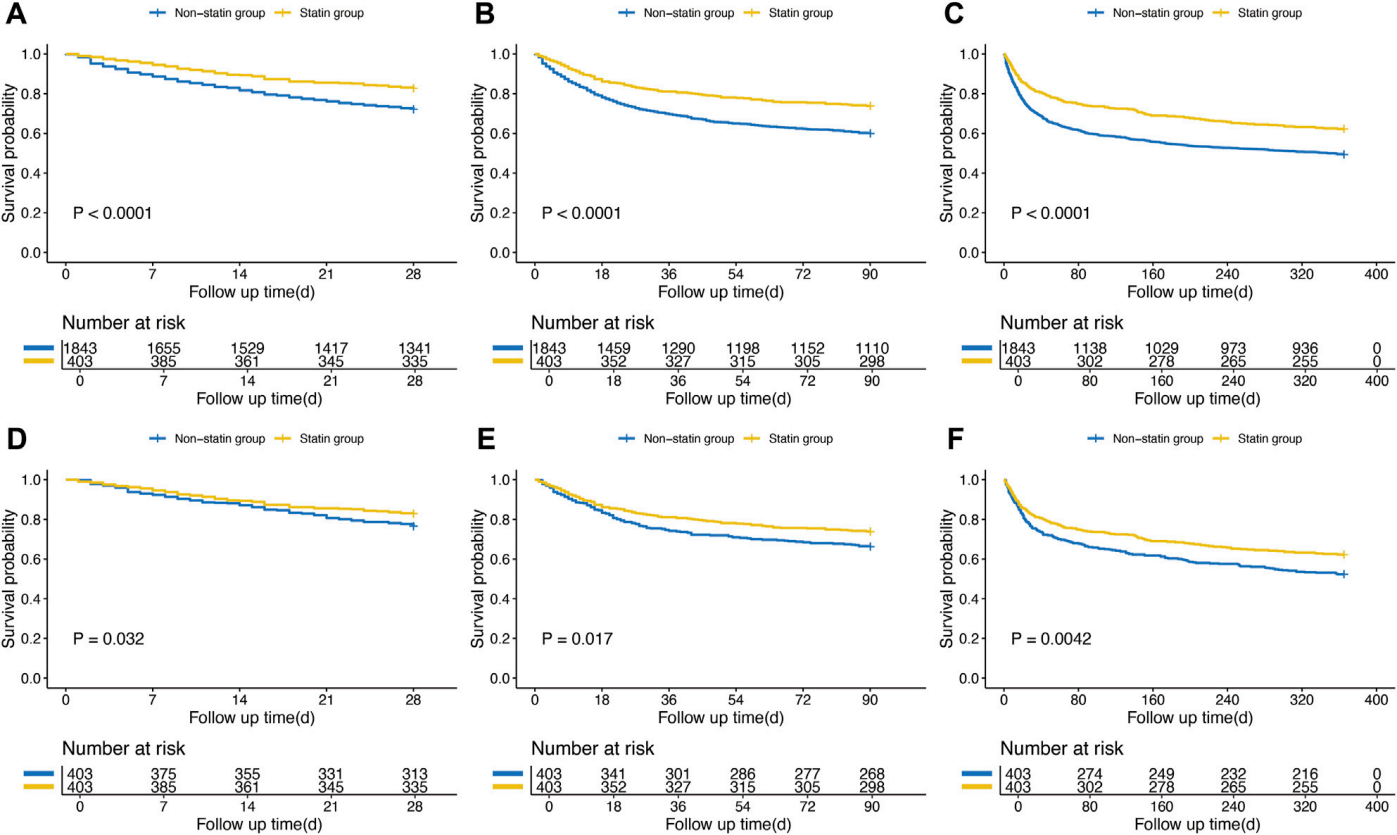
Kaplan-Meier survival curves of the non-statin group and statin group. **(A)** 28-day mortality before PSM; **(B)** 90-day mortality before PSM; **(C)** 1-year mortality before PSM; **(D)** 28-day mortality after PSM; **(E)** 90-day mortality after PSM; **(F)** 1-year mortality after PSM.

Differences in all-cause mortality rates were evaluated between the two groups during various follow-up periods: 28 days, 90 days, and 1 year. In the original cohort, the statin group exhibited significantly lower mortality rates at 28 days, 90 days, and 1 year compared to the non-statin group (P < 0.0001), as shown in Figure 2. After PSM, the Kaplan-Meier survival curves showed results consistent with those in the original cohort (P < 0.05). The baseline characteristics of the clinical outcomes of SIMI patients were shown in Supplementary Table S2.

### 3.3 The relationship between statin use and mortality

For the cohort following PSM, we further analyzed the association between statin administration and prognosis using Cox proportional hazard models, as shown in Table 2. In all three Cox proportional hazard models, statin use was closely associated with a reduced mortality risk among patients with SIMI. In the unadjusted Model I, statin use was associated with a 29% reduction in the risk of death at 28 days (HR = 0.71, 95% CI: 0.52–0.97, P = 0.032), a 27% reduction at 90 days (HR = 0.73, 95% CI: 0.57–0.95, P = 0.017), and a 27% reduction at 1 year (HR = 0.73, 95% CI: 0.60–0.90, P = 0.003). In Model II, which adjusted for vital signs and comorbidities, the 28-day mortality risk was reduced by 29% (HR = 0.71, 95% CI: 0.52–0.97, P = 0.030); the 90-day mortality risk decreased by 27% (HR = 0.73, 95% CI: 0.56–0.95, P = 0.018); and the 1-year mortality risk was reduced by 27% (HR = 0.73, 95% CI: 0.59–0.90, P = 0.004). In Model III, which included adjustments for biochemical indicators and treatment modalities, the 28-day mortality risk decreased by 32% (HR = 0.68, 95% CI: 0.49–0.94, P = 0.019); the 90-day mortality risk was reduced by 29% (HR = 0.71, 95% CI: 0.54–0.93, P = 0.012); and the 1-year mortality risk was lowered by 28% (HR = 0.72, 95% CI: 0.58–0.90, P = 0.003).

**TABLE 2:** Association between statin use and mortality.

Mortality	28-day mortality		90-day mortality		1-year mortality	
	HR (95%CI)	*P*	HR (95%CI)	*P*	HR (95%CI)	*P*
Model I	0.71 (0.52, 0.97)	0.032	0.73 (0.57, 0.95)	0.017	0.73 (0.60, 0.90)	0.003
Model II	0.71 (0.52, 0.97)	0.03	0.73 (0.56, 0.95)	0.018	0.73 (0.59, 0.90)	0.004
Model III	0.68 (0.49, 0.94)	0.019	0.71 (0.54, 0.93)	0.012	0.72 (0.58, 0.90)	0.003

HR, hazard ratio, CI, confidence interval.

Model I: crude.

Model II: Adjust: Age, Male, Race, HR, SBP, DBP, RR, SpO2, weight, Diabetes, Atrial Fibrillation, Cirrhosis, Hyperlipidemia, Hypertension, Obesity.

Model III: Adjust: Age, Male, Race, HR, SBP, DBP, RR, SpO2, weight, Diabetes, Atrial Fibrillation, Cirrhosis, Hyperlipidemia, Hypertension, Obesity, cTnT, CK-MB, HB, PLT, WBC, BUN, SCR, LAC, SOFA, SAPS II, LODS, IMV, CRRT, vasopressin, ACEI, ARB, aspirin, Beta-blockers, Fibrates.

We calculated that the E-Value for the effect of statin use on the one-year mortality rate for patients with SIMI is 1.82. This implies that any unmeasured confounding variable would need to have a relative risk association with both statin use and the one-year mortality rate from SIMI exceeding 1.82 for residual confounding to significantly affect the observed association. Using univariate Cox regression analysis, we determined the hazard ratios for factors strongly associated with SIMI mortality: atrial fibrillation (HR 1.40, 95% CI 1.20–1.80), liver cirrhosis (HR 1.60, 95% CI 1.10–2.40), hypertension (HR 0.76, 95% CI 0.61–0.93), and SOFA score (HR 1.10, 95% CI 1.00–1.10; see Supplementary Table S3). It is unlikely that unmeasured or unknown confounding factors would have a greater influence on SIMI mortality than the known risk factors, suggesting that their relative risk cannot exceed 1.82. Therefore, we conclude that unmeasured confounding factors are unlikely to have a substantial impact on our study results. Furthermore, the analysis of statin usage duration among SIMI patients revealed that the majority of patients initiated treatment within 24 h of ICU admission, as presented in the Supplementary Figure S2.

### 3.4 Association of statin type and dose with mortality

To further investigate the impact of various types and doses of statins on mortality rates in patients with SIMI, we excluded those who received multiple types or doses of statins during their hospitalization. We focused on patients administered a single type and dose of statin, excluding those treated with Lovastatin due to the limited number of records (only 2 instances). Ultimately, 376 patients with a single administration record were included for analysis.

The statins included in the analysis were atorvastatin (216 patients), simvastatin (108 patients), rosuvastatin calcium (23 patients), and pravastatin (29 patients). Survival analysis was performed using Cox proportional hazard models. The results of the Cox regression analysis for various statin medications are presented in Table 3. In our analysis of the relationship between different types of statins and mortality rates in patients with SIMI, we found that simvastatin was significantly associated with a reduction in mortality rates across multiple follow-up time points. Specifically, simvastatin demonstrated a significant reduction in mortality risk at 28 days (HR = 0.5, 95% CI: 0.28–0.90, P = 0.02), 90 days (HR = 0.5, 95% CI: 0.31–0.81, P = 0.005), and 1 year (HR = 0.6, 95% CI: 0.42–0.85, P = 0.004). However, there was no significant difference in the effect of atorvastatin, rosuvastatin calcium and pravastatin on short-term and long-term survival of SIMI. These results remained robust in Models II and III, which adjusted for multiple confounders, further confirming the potential benefits of statins in reducing both short-term and long-term mortality risks in patients.

**TABLE 3:** Association between different statins and mortality.

Mortality	28-day mortality		90-day mortality		1-year mortality	
	HR (95%CI)	*P*	HR (95%CI)	*P*	HR (95%CI)	*P*
Model I						
Non-statin	References					
Atorvastatin	0.88 (0.62, 1.26)	0.5	0.86 (0.64, 1.15)	0.3	0.79 (0.62, 1.02)	0.067
Pravastatin	0.55 (0.21, 1.50)	0.2	0.76 (0.38, 1.53)	0.5	0.81 (0.46, 1.42)	0.5
Rosuvastatin Calcium	0.17 (0.02, 1.15)	0.069	0.45 (0.17, 1.16)	0.1	0.62 (0.32, 1.18)	0.14
Simvastatin	0.5 (0.28, 0.90)	0.02	0.5 (0.31, 0.81)	0.005	0.6 (0.42, 0.85)	0.004
Model II						
Non-statin	References					
Atorvastatin	0.91 (0.63, 1.32)	0.6	0.89 (0.66, 1.21)	0.5	0.82 (0.63, 1.07)	0.14
Pravastatin	0.48 (0.17, 1.30)	0.15	0.7 (0.34, 1.44)	0.3	0.74 (0.41, 1.33)	0.3
Rosuvastatin Calcium	0.2 (0.03, 1.26)	0.087	0.52 (0.19, 1.39)	0.2	0.76 (0.41, 1.40)	0.4
Simvastatin	0.51 (0.27, 0.93)	0.029	0.5 (0.31, 0.82)	0.006	0.59 (0.41, 0.85)	0.005
Model III						
Non-statin	References					
Atorvastatin	0.83 (0.56, 1.22)	0.3	0.81 (0.59, 1.11)	0.2	0.77 (0.58, 1.01)	0.062
Pravastatin	0.5 (0.18, 1.35)	0.2	0.69 (0.33, 1.46)	0.3	0.65 (0.37, 1.13)	0.13
Rosuvastatin Calcium	0.34 (0.06, 1.89)	0.2	0.71 (0.28, 1.76)	0.5	0.96 (0.52, 1.75)	0.9
Simvastatin	0.5 (0.26, 0.98)	0.045	0.51 (0.31, 0.84)	0.008	0.6 (0.41, 0.87)	0.008

HR, hazard ratio, CI, confidence interval.

Model1: Crude.

Model2: Adjust: Age, Male, Race, HR, SBP, DBP, RR, SpO2, weight, Diabetes, Atrial Fibrillation, Cirrhosis, Hyperlipidemia, Hypertension, Obesity.

Model3: Adjust: Age, Male, Race, HR, SBP, DBP, RR, SpO2, weight, Diabetes, Atrial Fibrillation, Cirrhosis, Hyperlipidemia, Hypertension, Obesity, cTnT, CK-MB, HB, PLT, WBC, BUN, SCR, LAC, SOFA, SAPSII, LODS, IMV, CRRT, vasopressin, ACEI, ARB, aspirin, Beta-blockers, Fibrates.

### 3.5 Association of statin dose with mortality

To further investigate the impact of various doses of statins on mortality rates in patients with SIMI, we categorized statin use into low-dose and high-dose groups. High-dose statins were defined as atorvastatin 80 mg, simvastatin 80 mg, pravastatin 40 mg, and rosuvastatin calcium 20 mg per day (Pandit et al., 2016). Ultimately, 123 patients received high-dose statin therapy, whereas 253 patients were treated with low-dose statins. As shown in Table 4, the Cox proportional hazard models to examine the relationship between statin dosage and mortality revealed that low-dose statins were significantly associated with reduced 90-day and 1-year mortality risks. In the unadjusted model (Model I), the use of low-dose statins was significantly correlated with decreased mortality at 90 days (HR = 0.7, 95% CI: 0.52–0.94, P = 0.018), and 1 year (HR = 0.71, 95% CI: 0.56–0.90, P = 0.005). This risk reduction effect remained robust even after adjusting for multiple confounding factors in Models II and III. These results indicate that the use of low-dose statins may have significant clinical importance in improving patient outcomes.

**TABLE 4:** Association between different statin doses and mortality.

Mortality	28-day mortality		90-day mortality		1-year mortality	
	HR (95%CI)	P	HR (95%CI)	P	HR (95%CI)	P
Model I						
Non-statin	References					
Low dose	0.73 (0.51, 1.04)	0.079	0.7 (0.52, 0.94)	0.018	0.71 (0.56, 0.90)	0.005
High dose	0.64 (0.39, 1.04)	0.074	0.75 (0.51, 1.10)	0.14	0.74 (0.54, 1.02)	0.063
Model II						
Non-statin	References					
Low dose	0.72 (0.50, 1.03)	0.075	0.7 (0.51, 0.95)	0.021	0.7 (0.55, 0.90)	0.006
High dose	0.69 (0.42, 1.14)	0.15	0.8 (0.55, 1.18)	0.3	0.81 (0.59, 1.11)	0.2
Model III						
Non-statin	References					
Low dose	0.74 (0.51, 1.08)	0.12	0.71 (0.52, 0.97)	0.033	0.7 (0.54, 0.91)	0.008
High dose	0.57 (0.32, 1.03)	0.063	0.68 (0.45, 1.04)	0.076	0.74 (0.53, 1.04)	0.082

HR, hazard ratio, CI, confidence interval.

Model I: crude.

Model II: Adjust: Age, Male, Race, HR, SBP, DBP, RR, SpO2, weight, Diabetes, Atrial Fibrillation, Cirrhosis, Hyperlipidemia, Hypertension, Obesity.

Model III: Adjust: Age, Male, Race, HR, SBP, DBP, RR, SpO2, weight, Diabetes, Atrial Fibrillation, Cirrhosis, Hyperlipidemia, Hypertension, Obesity, cTnT, CK-MB, HB, PLT, WBC, BUN, SCR, LAC, SOFA, SAPSII, LODS, IMV, CRRT, vasopressin, ACEI, ARB, aspirin, Beta-blockers, Fibrates.

### 3.6 Subgroup analysis

**FIGURE 3:**
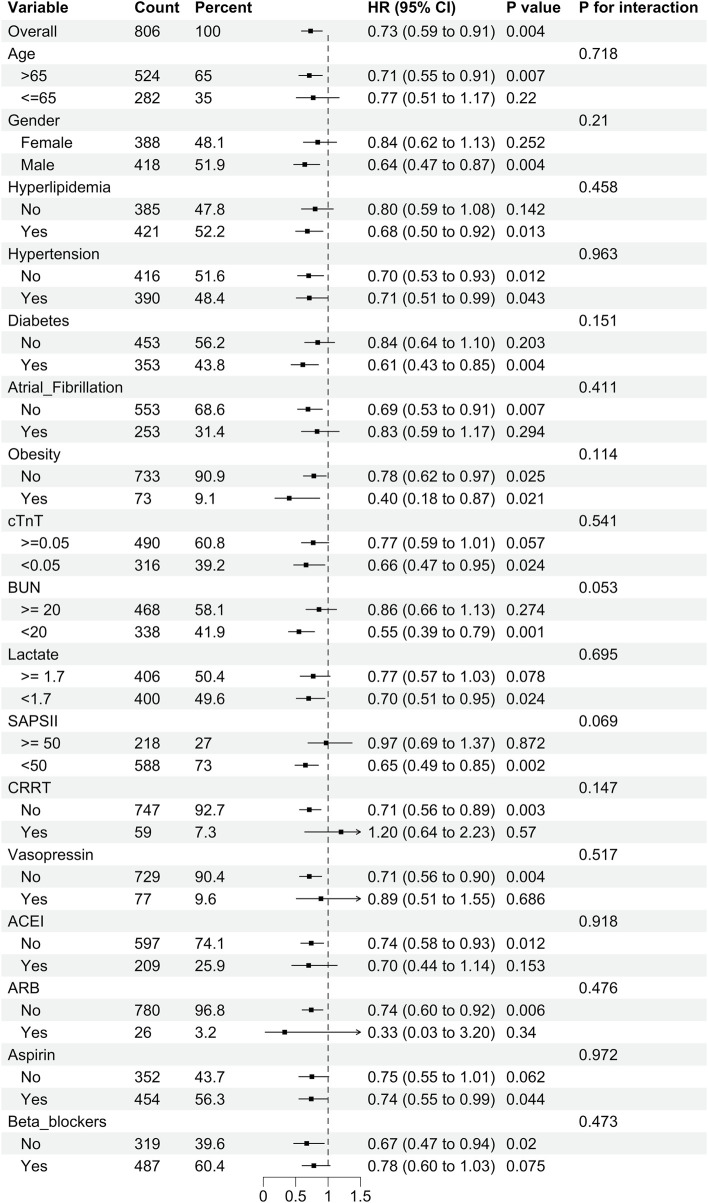
Subgroup analysis of the relationship between statin use and 1-year mortality in SIMI patients.

We conducted a subgroup analysis to explore the relationship between statin use and 1-year mortality, as shown in Figure 3. The results indicated no interaction between stratified variables and statin use (P for interaction >0.05).

Additionally, we analyzed the relationship between statin use and mortality at 28 days and 90 days across different subgroups (Supplementary Figure S3). The results indicated that in the subgroup analysis for 28-day mortality, there was no significant association between statin use and mortality in patients undergoing CRRT. However, in other subgroups, statin use appeared to influence patient outcomes to varying degrees. These findings support the potential benefits of statins in reducing both short-term and long-term mortality risks, with results remaining robust across different subgroups.

## 4 Discussion

This study utilized the MIMIC-IV v3.0 database to investigate the relationship between statin use and mortality risk in patients with SIMI. The results indicate a significant association between statin use and reduced short-term and long-term mortality risks in SIMI patients. Specifically, our findings demonstrate: (1) SIMI patients using statins experienced a 29%–32% reduction in 28-day mortality risk, a 27%–29% decrease in 90-day mortality risk, and a 27%–28% reduction in 1-year mortality risk compared to non-users; (2) Among statin types, simvastatin was associated with a 49%–50% reduction in the 28-day mortality risk, a 49%–50% reduction in the 90-day mortality risk, and a 40%–41% reduction in the 1-year mortality risk for patients with SIMI; (3) Regarding dosage, our findings revealed that low-dose statin use was significantly associated with reduced mortality risk compared to high-dose statin use.

Before this study, the association between statin use and mortality risk in patients with SIMI had not been examined. Our findings provide the first evidence that statin use is associated with improved outcomes in SIMI patients. However, the exact biological mechanisms underlying this relationship remain unclear. Research suggests that SIMI may involve various pathways, including the release of circulating myocardial depressant factors, downregulation of adrenergic pathways, production of nitric oxide and reactive oxygen species, abnormalities in calcium handling, mitochondrial dysfunction, impairment of coronary microvasculature, and downregulation of genes encoding myofibrillar and mitochondrial proteins (Hollenberg and Singer, 2021). Despite these insights, the pathogenesis of SIMI remains ambiguous, and effective therapeutic interventions to halt its progression are currently lacking (Beesley et al., 2018). Statins, commonly prescribed for cardiovascular diseases, play a vital role in treating atherosclerotic conditions by exerting varying degrees of regulatory effects on LDL, HDL, TG, and TC levels (Stancu and Sima, 2001; Delahoy et al., 2009; Yamashita et al., 2010; Ferraro et al., 2022). Prior studies have shown that statin use is associated with reduced mortality rates in patients with sepsis, although no correlation has been found with ICU or hospital length of stay (Li M. et al., 2024). Additionally, statin use may confer mortality benefits for patients with sepsis-induced coagulopathy (SIC) during their ICU stay (Yao et al., 2024). Additionally, statins have demonstrated anti-inflammatory properties and the potential for cardioprotection. Patients with a history of cardiovascular disease who receive statin therapy demonstrate lower rates of sepsis incidence and mortality (Rothberg et al., 2012). Statins may influence the sepsis process through multiple mechanisms, contributing to the mitigation of organ dysfunction by modulating inflammatory pathways and protecting cardiac tissue (Koushki et al., 2021). Research indicates that statins can inhibit inflammation and apoptosis in cells by decreasing the production of pro-inflammatory cytokines, such as TNF-α, IL-6, and IL-1β (de Jesus Oliveira et al., 2020). They also enhance antioxidant enzyme activity and reduce markers of oxidative stress in cardiac myocytes, thereby suppressing apoptosis in myocardial cells exposed to inflammatory stimuli (Jia et al., 2021). Additionally, statins can enhance endothelial function by increasing the bioavailability of nitric oxide, which is a vital molecule for vascular health. This improvement is linked to a reduction in oxidative stress and inflammation, with oxidative stress being a major contributor to endothelial dysfunction (German and Liao, 2023).

Our study further revealed that the type of statin utilized is associated with mortality risk, particularly in simvastatin, which showed significant effects. Existing research similarly suggests that different types of statins exert varying impacts on mortality in heart failure (Zheng et al., 2024). For instance, atorvastatin has demonstrated considerable efficacy regarding composite endpoints of all-cause mortality in adults with coronary artery disease (Lee et al., 2023). In patients with heart failure, lipophilic statins (e.g., atorvastatin and simvastatin) have shown more pronounced benefits in enhancing left ventricular ejection fraction and reducing fibrosis markers, such as sST2, compared to hydrophilic statins (e.g., rosuvastatin and pravastatin) (Bielecka-Dabrowa et al., 2019). Relevant experimental studies on septic myocardial dysfunction indicate that simvastatin may mitigate the inflammatory response in the hearts of rats with sepsis-induced myocardial depression by inhibiting the TLR4-NFκB pathway (Wang et al., 2017). Moreover, simvastatin has been shown to counteract endotoxin-induced apoptosis in cardiac myocytes and upregulate the expression of Survivin/NF-κB/p65 (Nežić et al., 2018). This may be attributed to the superior capacity of lipophilic statins to penetrate myocardial cells and their multifaceted effects on cardiac tissue (Techorueangwiwat et al., 2021). The results of our cohort study confirm this conclusion, showing a significant association between simvastatin, a lipophilic drug, and a reduction in SIMI mortality, whereas the role of hydrophilic statins was not statistically significant. In addition, the use of atorvastatin in SIMI patients in this study did not show a significant trend toward reduced mortality. Obviously, these results need to be verified in future studies, as they may be influenced by the number of patients using these drugs.

We identified a significant correlation between low-dose statin use and reduced mortality risk compared to high doses. This finding suggests that low doses may provide adequate protection during maintenance therapy while minimizing potential dose-related adverse effects. Currently, low-dose statins demonstrate substantial benefits in both primary and secondary prevention of atherosclerotic cardiovascular disease (ASCVD), with evidence supporting their efficacy across diverse populations (Barter, 2018). Low-dose statins are associated with a reduction in the incidence of cardiovascular mortality, acute coronary syndromes, and strokes (Thompson, 2016). In contrast, high-dose statins may be associated with more severe adverse effects. Research indicates an increased risk of elevated transaminases associated with high-dose statins, particularly atorvastatin, in the context of secondary prevention of cardiovascular disease (Wang et al., 2022). However, the benefits of high-dose statins in preventing cardiovascular events frequently outweigh these risks (Cai et al., 2021). Moreover, the potential for dose-dependent myotoxicity caused by statins should not be underestimated; the spectrum of myotoxicity associated with high-dose statins can vary from mild myopathy to severe rhabdomyolysis (du Souich et al., 2017). In our study, high-dose statins demonstrated a significant protective effect on 28-day mortality in adjusted Model III; however, in Model II and Model III, the protective effect of high-dose statins on 90-day and 1-year mortality was not statistically significant.

SIMI is a significant complication of sepsis and one of the leading causes of mortality among sepsis patients. Currently, there are no specific pharmacological agents available to intervene in the progression of SIMI (Martin et al., 2019). Our study is the first to investigate the impact of statin type, dosage, and lipid levels on both short-term and long-term mortality risks associated with SIMI. We anticipate that our findings will offer valuable insights for the clinical management of this serious condition.

Although this study identified a significant association between statin use and improved outcomes in patients with SIMI, several limitations must be acknowledged. First, this is a single-center retrospective cohort study utilizing the MIMIC-IV database. We found an association between statin use and reduced mortality in SIMI patients; however, causality cannot be established from our findings. Second, the study population focuses on SIMI, which does not cover all subtypes of sepsis, which may limit the generalizability of the results. Third, there is currently no consensus on the diagnostic criteria for SIMI. The database contained numerous missing values for key indicators, such as NT-proBNP, hemodynamic data and echocardiography results, prompting us to adopt cTnT >0.01 ng/mL as the diagnostic criterion based on prior studies and the reference upper limit in the database. Fourth, although we employed the PSM method and E-Value analysis indicating that confounding factors are unlikely to significantly affect the outcomes, the design of the study inherently implies the presence of confounding factors. Fifth, due to a significant amount of missing data for laboratory parameters such as LDL and TC in the database, we were unable to incorporate lipid profiles into the baseline statistics and outcome measures. Sixth, the diagnosis of comorbidities in this study was based on ICD codes for discharge diagnoses recorded in the MIMIC-IV database, and the exact admission diagnoses could not be obtained. Seventh, this study focused solely on the type and dosage of statins administered during hospitalization, with no data available on post-discharge medication, which may impact the results. These limitations may have influenced the results of this study, and therefore the data should be interpreted with caution. We suggest that future studies should address these limitations and could explore the role and safety of different types and doses of statins in the treatment of SIMI under the definition of SIMI in more dimensions, preferably with larger sample sizes and long-term follow-up studies.

## 5 Conclusion

In conclusion, our study indicates a significant association between statin use and decreased mortality in patients with SIMI at 28 days, 90 days, and 1 year. Notably, simvastatin demonstrates considerable benefits, with low-dose statins providing greater advantages compared to high-dose formulations. These findings advocate for the incorporation of statins into treatment strategies for patients with SIMI.

## Data Availability

The datasets presented in this study can be found in online repositories. The names of the repository/repositories and accession number(s) can be found below: https://physionet.org/content/mimiciv/3.0/.
