# Supplementary material for: Impact of statin use on short- and long-term outcomes in patients with sepsis-induced myocardial injury: insights from the MIMIC-IV database
Source: Front Pharmacol. 2025 Mar 19;16:1520107. doi: 10.3389/fphar.2025.1520107 (PMC11962036; doi:10.3389/fphar.2025.1520107)
Supplement: Supplementary file 1 [file DataSheet1.docx]

Supplementary Material

## Supplementary Figures

**
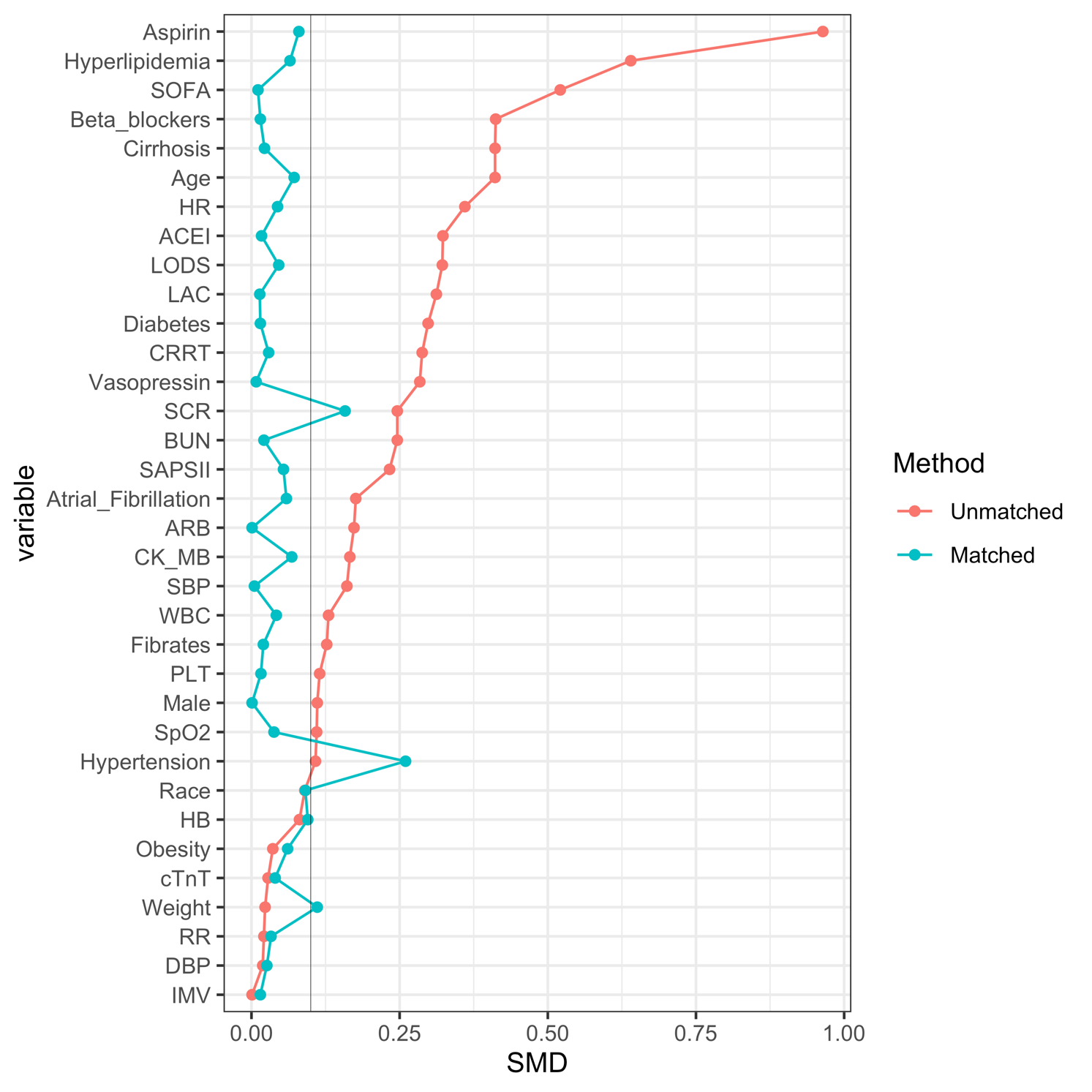
**

**Supplementary Figure S1**. Comparison of standardized mean differences (SMD) before and after propensity score matching (PSM).

**
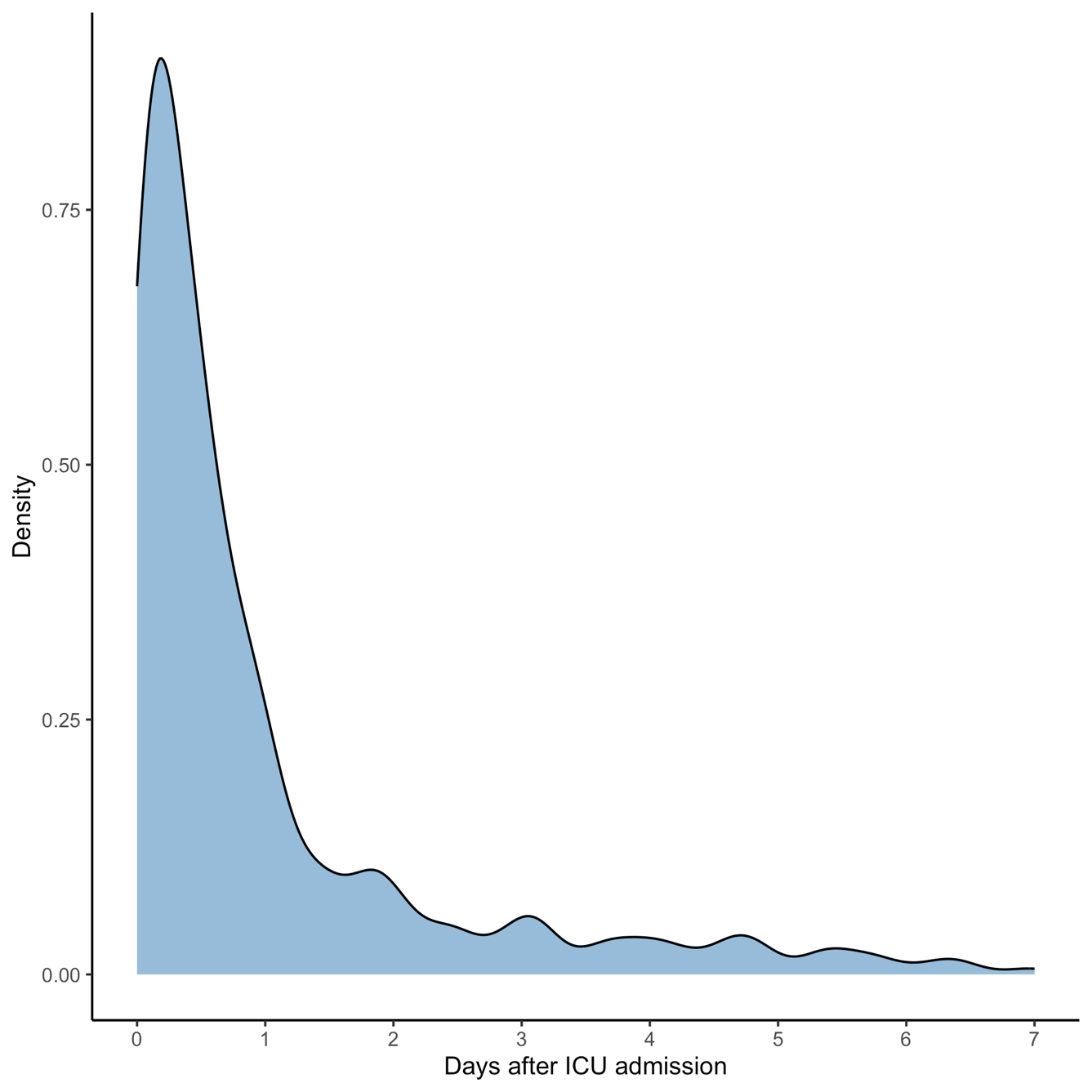
**

**Supplementary Figure S2**. Kernel density plot of time to statin initiation.


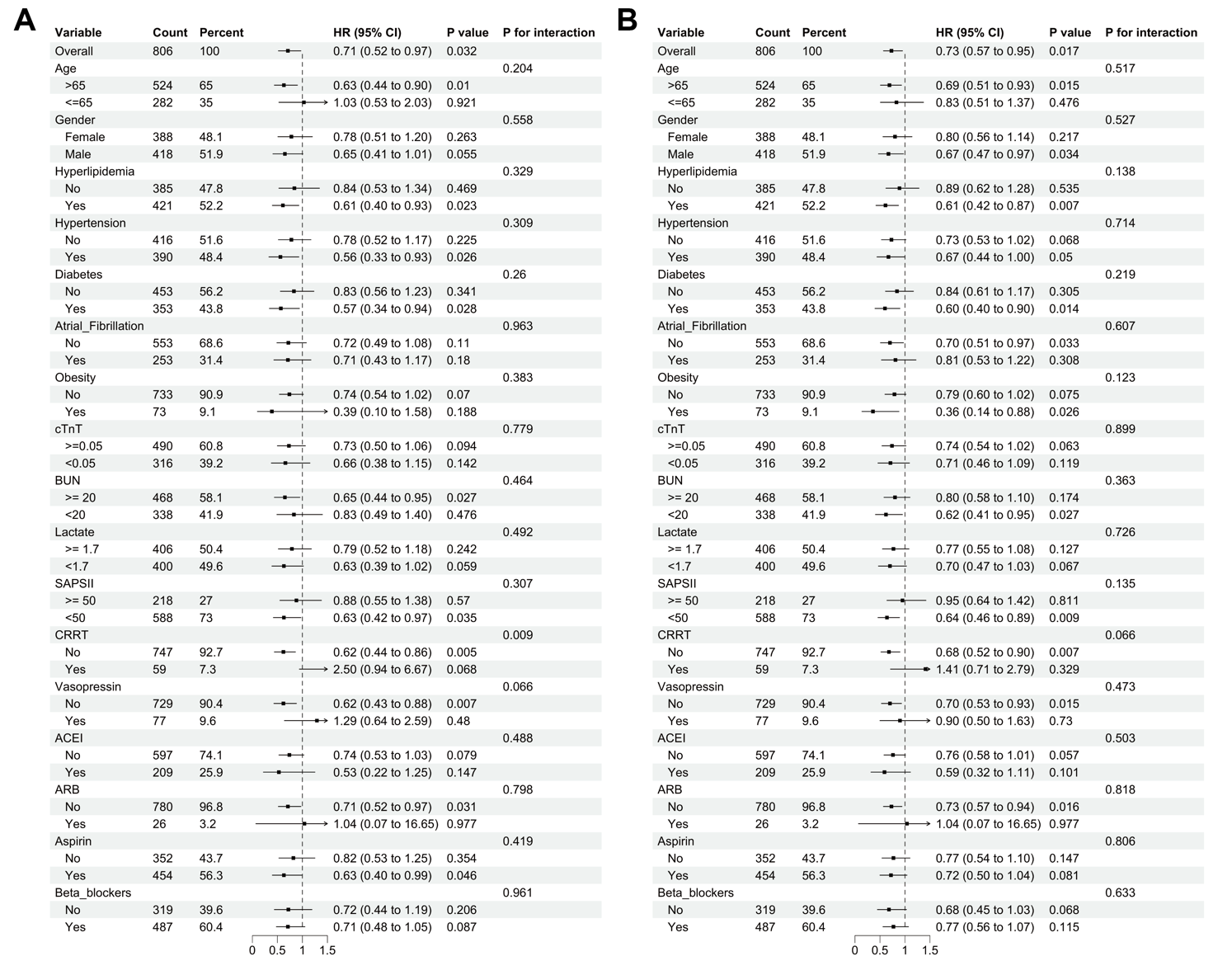


**Supplementary Figure S3**. Subgroup analysis of the relationship between statin use and 28-day (A) and 90-day (B) mortality in sepsis-induced myocardial injury (SIMI) patients.

## Supplementary Tables

| **Variable** | **Missing Percent (%)** |
| --- | --- |
| LDL | 98.71 |
| NT-pro BNP | 87.44 |
| HDL | 86.91 |
| CRP | 85.35 |
| TC | 85.31 |
| TG | 64.16 |
| Height | 43.46 |
| CK | 39.58 |
| CK MB | 28.63 |
| LAC | 18.97 |
| WBC | 7.44 |
| HB | 6.81 |
| PLT | 6.23 |
| Weight | 1.91 |
| SCR | 0.31 |
| RR | 0.27 |
| BUN | 0.27 |
| HR | 0.22 |
| SBP | 0.22 |
| DBP | 0.22 |
| SpO2 | 0.22 |
| Age | 0 |
| Male | 0 |
| Race | 0 |
| Diabetes | 0 |
| Atrial Fibrillation | 0 |
| Cirrhosis | 0 |
| Hyperlipidemia | 0 |
| Hypertension | 0 |
| Obesity | 0 |
| cTnT | 0 |
| SOFA | 0 |
| SAPSII | 0 |
| LODS | 0 |
| IMV | 0 |
| CRRT | 0 |
| Vasopressin | 0 |
| ACEI | 0 |
| ARB | 0 |
| Aspirin | 0 |
| Beta-blockers | 0 |
| Fibrates | 0 |
| statins | 0 |
| LOS ICU days | 0 |
| LOS hospital days | 0 |
| Hospital mortality | 0 |
| 28-day mortality | 0 |
| 90-day mortality | 0 |
| 1-year mortality | 0 |

**Supplementary Table S1**. Missing number for included variables in the datasets.

| **Characteristic** | **Original cohort** | | | **PSM cohort** | | | |
| --- | --- | --- | --- | --- | --- | --- | --- |
|  | **Non-statin group** | **Statin group** | ***P***^1^ | **Non-statin group** | | **Statin group** | ***P***^1^ |
|  | N = 1,843 | N = 403 |  | N = 403 | | N = 403 |  |
| **Length of Stay (LOS), Median (Q1, Q3)** | | | | | | | |
| LOS ICU days | 4.28 (2.26, 9.92) | 4.13 (2.22, 8.92) | 0.392 | 4.32 (2.32, 9.65) | | 4.13 (2.22, 8.92) | 0.556 |
| LOS hospital days | 13.80 (6.73, 27.22) | 10.71 (6.90, 17.81) | <0.001 | 13.81 (6.59, 24.20) | | 10.71 (6.90, 17.81) | 0.011 |
| **Outcomes, n (%)** | | | | | | | |
| Hospital mortality | 519.00 (28.16%) | 60.00 (14.89%) | <0.001 | 77.00 (19.11%) | | 60.00 (14.89%) | 0.111 |
| 28-day mortality | 513.00 (27.84%) | 69.00 (17.12%) | <0.001 | 94.00 (23.33%) | | 69.00 (17.12%) | 0.028 |
| 90-day mortality | 737.00 (39.99%) | 105.00 (26.05%) | <0.001 | 136.00 (33.75%) | | 105.00 (26.05%) | 0.017 |
| 1-year mortality | 932.00 (50.57%) | 152.00 (37.72%) | <0.001 | 192.00 (47.64%) | | 152.00 (37.72%) | 0.004 |
| ^1^Wilcoxon rank sum test; Pearson's Chi-squared test | | | | |  | | |

**Supplementary Table S2**. Clinical outcomes before and after PSM population.

| **Characteristic** | **HR (95%CI)** | **P** |
| --- | --- | --- |
| Age | 1.00 (1.00-1.00) | <0.001 |
| Male | 0.88 (0.71-1.10) | 0.22 |
| Race | 0.88 (0.77-1.00) | 0.057 |
| HR | 1.00 (1.00-1.00) | 0.021 |
| SBP | 1.00 (0.99-1.00) | 0.2 |
| DBP | 0.99 (0.98-1.00) | 0.21 |
| RR | 1.00 (0.99-1.00) | 0.21 |
| SpO2 | 0.99 (0.98-1.00) | 0.32 |
| Weight | 0.99 (0.99-1.00) | <0.001 |
| Diabetes | 0.81 (0.65-1.00) | 0.05 |
| Atrial Fibrillation | 1.40 (1.20-1.80) | 0.001 |
| Cirrhosis | 1.60 (1.10-2.40) | 0.019 |
| Hyperlipidemia | 0.87 (0.71-1.10) | 0.2 |
| Hypertension | 0.76 (0.61-0.93) | 0.01 |
| Obesity | 0.80 (0.54-1.20) | 0.27 |
| cTnT | 1.00 (0.83-1.20) | 0.97 |
| CK MB | 1.00 (1.00-1.00) | 0.86 |
| HB | 0.93 (0.87-0.99) | 0.03 |
| PLT | 1.00 (1.00-1.00) | 0.005 |
| WBC | 1.00 (1.00-1.10) | <0.001 |
| BUN | 1.00 (1.00-1.00) | <0.001 |
| SCR | 0.98 (0.93-1.00) | 0.46 |
| LAC | 1.10 (1.00-1.20) | 0.009 |
| SOFA | 1.10 (1.00-1.10) | <0.001 |
| SAPSII | 1.00 (1.00-1.00) | <0.001 |
| LODS | 1.10 (1.10-1.10) | <0.001 |

**Supplementary Table S3**. Risk factors for 1-year mortality in patients with SIMI identified by univariate cox regression.
